# Prevalence of Malocclusions in Down Syndrome Population: A Cross-Sectional Study

**DOI:** 10.3390/medicina59091657

**Published:** 2023-09-14

**Authors:** Anna Alessandri-Bonetti, Federica Guglielmi, Antongiulia Mollo, Linda Sangalli, Patrizia Gallenzi

**Affiliations:** 1 Institute of Dental Clinic, A. Gemelli University Policlinic IRCCS, Catholic University of Sacred Heart, 00168 Rome, Italy; anna.alessandribonetti01@icatt.it (A.A.-B.); federica.guglielmi01@icatt.it (F.G.); antongiulia.mollo01@icatt.it (A.M.); patrizia.gallenzi@unicatt.it (P.G.); 2 College of Dental Medicine—Illinois, Midwestern University, 555 31st Street, Downers Grove, IL 60515, USA

**Keywords:** down syndrome, malocclusions, angle relationship, orthodontic parameters

## Abstract

*Background and Objectives*: A higher prevalence of dental malocclusion has been suggested among individuals with Down Syndrome (DS) compared to controls, although no studies to date have investigated such a difference according to age group. Therefore, the aim of this study was to compare the prevalence of dental malocclusion and other orthodontic measures between DS and non-syndromic (non-DS) individuals across three age groups of children, adolescents, and adults. *Materials and Methods*: This cross-sectional study was conducted on a total of 147 patients. Of those, 72 were diagnosed with DS and were divided into N = 15 children (<10 years), N = 23 adolescents (10–18 years) and N = 34 adults (>18 years). The remaining 75 patients were sex- and age-matched controls. The two groups were compared according to age group in terms of the prevalence of dental malocclusion, measures of sagittal, vertical, and transverse discrepancy, facial profile, and probable sleep bruxism with chi-square tests for proportion. *Results*: The DS patients consistently exhibited a higher prevalence of Class 3 malocclusion, concave profile and anterior crossbite compared to the non-DS patients, regardless of age group. The non-DS adolescents presented with a significantly higher prevalence of convex profile than the DS adolescents. The adolescent and adult DS patients most commonly presented with a maxillary transverse discrepancy and posterior crossbite compared to the non-DS controls. The DS adults had a higher prevalence of probable sleep bruxism. *Conclusions*: Patients with DS showed a higher prevalence of Class 3 malocclusion, concave profile and anterior crossbite compared to non-DS controls, regardless of age group. Other orthodontic measures showed a fluctuation according to the age group considered.

## 1. Introduction

Trisomy 21, also called Down Syndrome [1], is the most common chromosome abnormality in newborns [2], and it is caused by the presence of an additional third copy of chromosome 21. Children with DS experience various degrees of intellectual disability and are more vulnerable to health complications, including hearing problems, respiratory disease, and vision impairment, among many other congenital abnormalities [3,4,5,6,7,8,9]. Limited to the craniofacial area, a growing body of literature has shown that children and adolescents with DS more commonly exhibit craniofacial and dentoalveolar abnormalities compared to healthy controls. Among these, posterior crossbite, Angle Class 3 malocclusions and anterior open bite have been the most commonly reported [10]. To date, most of the available studies have been conducted on growing individuals. However, the data obtained in younger individuals may not be indicative of the DS population when the growth is completed, considering the significant occlusal changes that occur between primary [11] and permanent dentition which sometimes lead to spontaneous improvements of these parameters [12]. Moreover, the mandibular growth ends around the age of 17 in females and 19 in males [13], and data obtained before this age may not reflect the adult population. To the best of our knowledge, malocclusion in the adult DS population has never been evaluated. Moreover, it is known that ethnic traits may influence the prevalence and characteristics of malocclusion [3]. Therefore, population-specific studies are essential for tailoring healthcare interventions, adopting preventive measures to address the unique local needs and informing resource allocation for conditions specific to that ethnic or racial consideration. Moreover, epidemiological research focused on a specific population may further elucidate to what extent genetic factors can play a role in the etiology of the condition and how these traits interact with environmental influences.

Therefore, the primary aim of the present study was to determine the prevalence of malocclusion in an Italian DS patient population compared to non-syndromic (non-DS) individuals by age group. It was hypothesized that individuals diagnosed with DS will exhibit malocclusion more frequently than the non-DS controls, regardless of age group. A secondary aim was to investigate the differences in the orthodontic parameters between the DS and non-DS patients by age group [5,6]. As this aim was exploratory in nature, no a-priori hypothesis was formulated. 

## 2. Materials and Methods

This cross-sectional study was conducted at the Dental Clinic for Pediatric and Special Needs patients of the Institute of Dental Clinic of the A. Gemelli University Policlinic IRCCS (Catholic University of Sacred Heart, Rome, Italy). This study was conducted in full conformance with the principles of the “Declaration of Helsinki,” and good clinical practice. The research was approved by the Ethics Committee of the university where the study was conducted (Protocol ID 4221).

### 2.1. Participants

A sample size calculation was performed based on the primary aim of the study of assessing the prevalence of malocclusion in the DS population compared to the non-DS population. The sample size was calculated assuming 74.5% of malocclusions in the DS population and 14% in non-DS population [14]. Considering a 95% confidence interval and a power of 0.80, the minimum sample size required to obtain a statistically significant difference was 11 participants per group.

All consecutive DS patients referred to the dental clinic were invited to participate in the study, after an extensive explanation was provided by one of the study personnel. DS patients were allocated in three groups based on their age: those <10 years old were classified as *children*; those between 10 and 18 years old were classified as *adolescents*; those ≥18 years old were classified as *adults*. These age groups were based on the literature that indicated class 3 as the most common malocclusion in DS subjects [15]. As such, the cut-off of 10 years of age to identify *children* was selected because in cases of untreated Class 3 malocclusion, the mandibular spurt occurs between 10 and 12 years of age [16]. The cut-off of 18 years was selected because after 18 years the mandibular growth rate is suggested to decrease significantly [13,17] and the growth in posterior cranial base to be completed [18].

For the non-DS group, sex- and age-matched non-syndromic patients were selected from dental patients referred to the dental clinic for a first evaluation. The non-DS group was similarly divided into three age groups, as described above.

Exclusion criteria of both groups included a history of previous orthodontic treatment, which might have potentially influenced the development or correction of an initial malocclusion; a history of jaw injuries or trauma, which could result in misalignment and malocclusion; radiation therapy for head and neck cancers, which might have affected jaw development; the presence of coexisting syndromes that could contribute to the occurrence of dental malocclusion (e.g., cleft palate, Crouzon syndrome, Treacher Collins syndrome, Marfan syndrome); severe cognitive impairment, behavioral challenges, or intellectual disabilities, that could impede the dental examination process; pregnancy state, as hormonal changes during pregnancy could affect oral health; and a lack of signed informed consent.

### 2.2. Procedures

After obtaining a signed informed consent by the patients and their legal guardians, all DS patients underwent an assessment of their anamnestic data and a complete dental and orthodontic evaluation performed by the same trained examiner. The guardians of the patients were present during the entire evaluation.

### 2.3. Measures

The following sagittal, transversal, and vertical measures were collected during the clinical examination:

− ***malocclusion***: the type of malocclusion was assessed by recording the molar class relationship based on Angle’s classification of malocclusion, and classified in Angle’s Class 1, Class 2 or Class 3 malocclusion [19].
− ***maxillary transverse discrepancy***: this was evaluated by evaluating the form, symmetry of the maxillary arch, palatal vault shape, predominant breathing mode (i.e., oral or nasal) and buccal corridor width when smiling [20]. The palatal transverse discrepancy was classified as posterior crossbite when the buccal cusps of the upper molars were in contact with the central fossae of the lower molars [21]. It was classified as scissor bite in the presence of a posterior discrepancy with or without contact between the palatal surface of the upper lingual cusp and the buccal surface of the lower buccal cusp [22].
− ***sagittal discrepancy***: anterior crossbite was defined as the presence of a negative horizontal overlap between upper and lower incisors, measured from the facial surface of the upper incisors to the middle of the incisal edge of the lower incisors [23].
− ***dental crowding***: this was measured using the Little’s irregularity index, a tool to estimate the difference between available and required space [24].
− ***vertical discrepancy***: the amount of vertical overlapping of upper and lower incisors was calculated, considering a value of 4 mm as the cut-off for presence of anterior deep bite [25]. Conversely, anterior open bite was defined as the presence of a negative vertical overlapping between upper and lower incisors [23].
− ***facial profile***: the patient’s profile was classified as concave, straight and convex based on the definition provided by Arnett and Bergman [26].
− Finally, the presence of ***probable sleep bruxism*** was ascertained according to the grading system proposed by the international consensus [27]. As such, a positive response was assigned based on patient/guardian report and on the presence of at least one clinical sign (e.g., masticatory muscle hypertrophy, buccal linea alba, indentations on the tongue or lip, tooth wear) [27].

### 2.4. Statistical Analysis

Data were summarized using descriptive statistics as mean and standard deviation, or median and interquartile range (for continuous variables), and as frequency distribution (for categorical variables). Normality distribution was evaluated through a Shapiro-Wilk test, and skewed variables were transformed as needed. To test the primary aim (i.e., assessing the difference in prevalence of malocclusion between DS and non-DS individuals by age group), DS patients and non-DS patients belonging to each age group were compared with chi-square tests for proportion. To test the secondary aim (i.e., assessing the difference in orthodontic parameters between DS and non-DS individuals by age group), all the orthodontic parameters were dichotomized and assigned a value of 1 in case of presence, and a value of 0 in case of absence. Then, DS patients were compared with non-DS patients by age group in the selected orthodontic parameters with chi-square tests.

For all analyses, the *p*-value was set at <0.05. Data were analyzed using SPSS (IBM SPSS Statistics Macintosh, Version 27.000, IBM Corp., Armonk, NY, USA).

## 3. Results

A total of 147 patients were recruited for the study. Seventy-two DS patients (64% males, average age of 18.9 ± 11.1) were included and compared with 75 sex- and age-matched non-DS patients.

### 3.1. Children Population (<10 y.o.)

**Figure 1:**
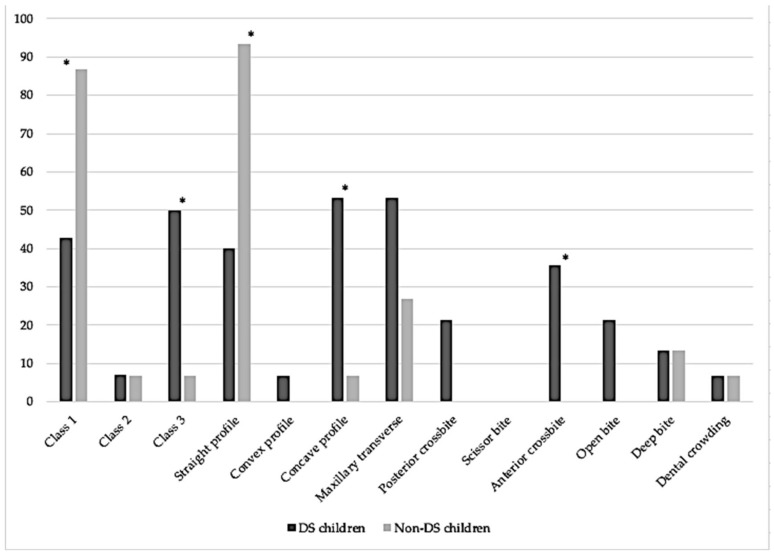
Bar charts display the difference between DS and non-DS children in prevalence of malocclusion and orthodontic parameters. * identifies statistically significant differences at *p* < 0.05.

A total of 15 DS children (80% males, average age 6.7 ± 2, range between 4 and 10) were compared with 15 sex- and age-matched controls. The DS children presented with a statistically significantly lower prevalence of Angle’s Class 1 malocclusion (*p* = 0.021) and straight profile (*p* = 0.005), and a higher prevalence of Angle’s Class 3 malocclusion (*p* = 0.014), concave profile (*p* = 0.014) and anterior cross bite (*p* = 0.017). There was no statistically significant difference in any of the remaining orthodontic parameters. The prevalence of probable sleep bruxism did not differ between the two groups. Table 1 and Figure 1 present the difference in the malocclusion and orthodontic parameters between the DS and non-DS children.

### 3.2. Adolescent Population (10–17 y.o.)

**Figure 2:**
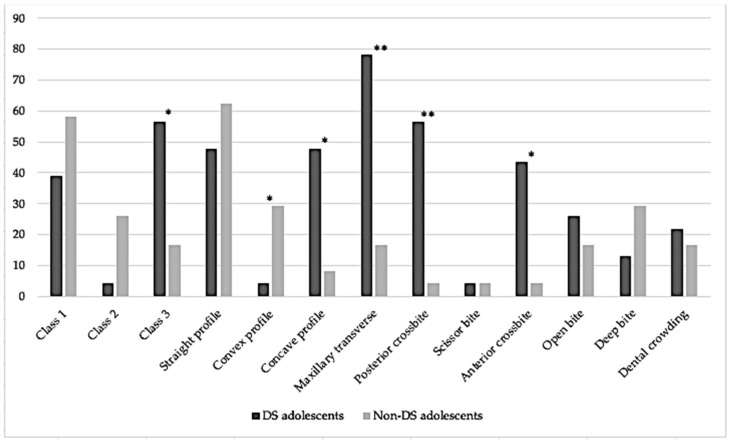
Bar charts display the difference between DS and non-DS adolescents in prevalence of malocclusion and orthodontic parameters. * identifies statistically significant differences at *p* < 0.05. ** identifies statistically significant differences at *p* < 0.001.

DS adolescents (N = 23, 47.8% males, average age 13.13 ± 2.3, range between 10 and 17) were compared to sex and age matched controls. The DS adolescents presented with a significantly higher prevalence of Angle’s Class 3 malocclusion (*p* = 0.006), concave profile (*p* = 0.003), palatal transverse discrepancy (*p* < 0.001), posterior crossbite (*p* < 0.001) and anterior cross bite (*p* = 0.002). The non-DS adolescents presented with a higher prevalence of convex profile compared to the DS adolescents (*p* = 0.048). No other statistically significant difference was found in any of the remaining orthodontic parameters. Table 2 and Figure 2 present the difference in the malocclusion and orthodontic parameters between the DS and non-DS children.

### 3.3. Adult Population (≥18 y.o.)

**Figure 3:**
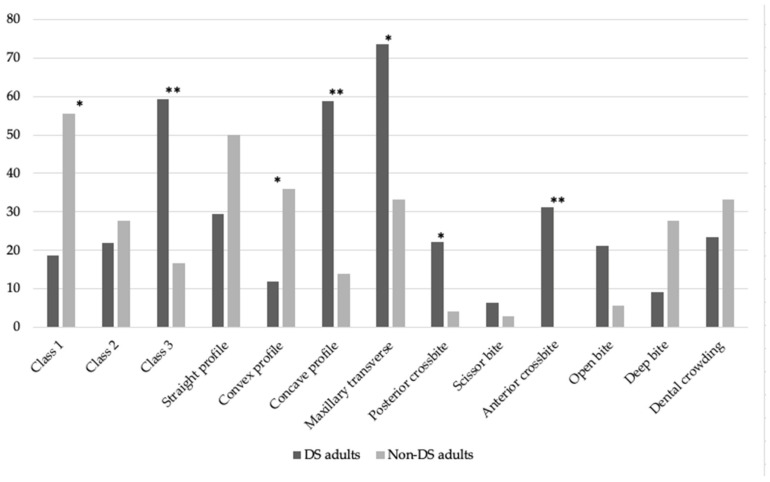
Bar charts display the difference between DS and non-DS adults in prevalence of malocclusion and orthodontic parameters. * identifies statistically significant differences at *p* < 0.05. ** identifies statistically significant differences at *p* < 0.001.

DS adults (N = 34, 67.6% males, average age 28.1 ± 9, range between 18 and 51 y.o) were compared to sex- and age-matched controls. The DS adults presented with a significantly lower prevalence of Angle’s Class 1 malocclusion (*p* = 0.003) and convex profile (*p* = 0.025), and a higher prevalence of Angle’s Class 3 malocclusion (*p* < 0.001), concave profile (*p* < 0.001), palatal transverse discrepancy (*p* = 0.001), posterior crossbite (*p* = 0.001), anterior cross bite (*p* < 0.001) and probable sleep bruxism (*p* = 0.002). No other orthodontic parameters differed between the two groups. Table 3 and Figure 3 present the difference in the malocclusion and orthodontic parameters between the DS and non-DS adults.

## 4. Discussion

The present study aimed to assess the prevalence of malocclusion in an Italian DS patient population compared to non-syndromic age- and sex-matched controls by analyzing the difference according to age groups. To the best of our knowledge, this is the first study examining the prevalence of malocclusion and orthodontic parameters in a DS patient population across different age groups.

The results of this study revealed that Class 3 malocclusion was the most commonly observed malocclusion in the DS group, with a progressively increasing trend from childhood (50.0% vs. 6.7%, *p* = 0.014) to adulthood (59.4% vs. 16.7%, *p* < 0.001). A high prevalence of Class 3 malocclusion among DS individuals had already been suggested by previous studies [3], although no data have been recorded according to different age group populations. The increase in prevalence of Class 3 and Class 2 malocclusion in orthodontically untreated patients over time advocates for early orthodontic treatment, which may consist of face-mask and rapid palatal expansion for Class 3, or functional appliance for Class 2, among others. As for the correct timing of intervention, a recent meta-analysis on the effectiveness of maxillary protraction during different stages of dentition failed to reveal any difference in achieving successful outcomes if the treatment is applied in late-mixed or early-mixed dentition [28]. Taken together, these findings provide a theoretical basis for extending the applicable age period of maxillary protraction, thus allowing clinicians to better tailor the orthodontic therapy to the patient’s needs.

Our results showed an increasing prevalence of palatal transverse discrepancy within growing DS patients (from 53.3% in children to 78.3% in adolescents and 73.5% in adults). Conversely, a reduction in prevalence was observed in the non-DS group from childhood to adolescence (26.7% vs. 16.7%). These findings are in accordance with a recent study showing that in orthodontically untreated non-DS children, a spontaneous correction of the palatal discrepancy and posterior crossbite can be expected with growth [29]. However, it also emphasizes an important difference between DS and non-DS patients. As it is established that DS patients commonly experience sleep-disordered breathing [30,31], the presence of obstructive sleep apnea (OSA) and its close connection to mouth breathing due to adenotonsillar hypertrophy in children might have been a significant factor contributing to the development and maintenance of malocclusion. Unfortunately, the expense associated with a polysomnography (PSG) examination for a conclusive diagnosis of OSA prevented us from investigating sleep-disordered breathing conditions in the present study.

As PSG was not conducted, the evaluation of sleep bruxism relied on the grading system proposed by Lobbezoo et al. in the International Consensus [27]. Accordingly, a positive assessment indicating probable sleep bruxism was established through a patient/parent self-report and positive inspection during clinical examinations. Of note, a positive clinical examination assessment has demonstrated good accuracy and sensitivity (72.0% and 70.8%, respectively) in comparison to a home-recorded device, while a positive response regarding tooth grinding within the past two weeks from a bed partner or others has shown excellent specificity (96.8%) [32]. In the present study, a statistically significant difference was found between DS vs. non-DS adults (*p* = 0.002). Although the difference between the two groups was not statistically significant across the other age categories (*p* = 0.272 in children and *p* = 0.060 in adolescents), a higher prevalence of probable sleep bruxism was found in the DS patients, while a fluctuation was observed in the non-DS patients, with a higher peak during childhood. Interestingly, a recent review investigating sleep bruxism in DS children suggested that the prevalence may vary significantly in different countries, and reported the prevalence in the Italian sample to be consistent with our results [33].

For the current study, an Italian population was selected. Population-specific studies are important as they allow healthcare providers and researchers to tailor treatments and preventive measures to address the specific needs of the local population. This approach enhances the translational applications of epidemiological research. In the context of this study, understanding the prevalence and characteristics of malocclusion in Italian individuals can assist orthodontists and dentists in developing more effective treatment plans and proactively anticipating common issues, with the ultimate goal of improving the oral health and quality of life of their patients. Similarly, the findings from this study can guide orthodontic intervention strategies within the Italian DS population. This proactive approach has the potential to yield improved outcomes and reduce the severity of malocclusion. Moreover, population-specific epidemiological research can inform healthcare planning and resource allocation. This includes securing funding for specialized dental care, orthodontic services and public health initiatives targeting individuals with DS. Exploring epidemiological trends in the Italian DS population may also shed light on how genetic factors specific to the Italian population interact with environmental factors, potentially influencing the development of malocclusion. These insights can contribute to a broader understanding of the condition’s etiology.

Comparative studies across different populations can help researchers identify universal patterns and population-specific trends in malocclusion. When comparing the results of the present study with the available literature from other countries, a similar prevalence of malocclusion was found in DS patients in Saudi Arabia [34], while contraposing results were observed for non-DS subjects. This may suggest that the ethnic inheritance in craniofacial features and malocclusions [35] tends to play only a marginal role in the DS population [3,36], while being primarily supported by the genetic syndromic characteristics. Oliveira et al. identified factors such as age, parafunctional activities (*e.g*., nail or finger biting), mouth breathing and sore-throat episodes to be associated with a higher prevalence of malocclusions in DS subjects [37]. Similarly, the present study confirmed that age is an important factor; however, data regarding oral parafunctional habits or other conditions were not assessed.

### Limitations

The current study has some limitations. The main limitation lies in the fact that the current findings are specific to the age grouping used in this study. Various classifications suggest different age cut-offs to differentiate children from adolescents. For instance, the American Medical Association suggests an age of 12 [38], while Statistics Canada proposes an age of 14 [39]. However, a threshold of 10 was selected in this study to distinguish children from adolescents based on the growth spurt of the mandibular bone. Despite this choice, the distribution of the participants between the two groups (DS vs. non-DS) was homogeneous across age groups, ensuring that the comparison between the groups remains reliable and valid across the different age categories.

The objective of the present research—to investigate the difference in orthodontic parameters between DS and non-DS patients—was achieved by dichotomizing the variables. This approach is known to decrease the power of the analysis and to allow for the correction of confounding factors only partially [40]. Nevertheless, the difference between the groups was significant in almost all the considered orthodontic measures. As such, we foresee that a comparison between continuous variables would have most likely achieved the same results. Another limitation is the cross-sectional nature of the study, which does not allow for any longitudinal follow-up of the participants.

Moreover, as patients were excluded if they reported previous orthodontic treatments, it is not possible to predict the impact of a potential orthodontic therapy. In addition, the study was conducted on patients referred to a dental clinic. As such, it may not be representative of the complete patient population. Another limitation consists in the fact that the assessment of cross-bite malocclusion did not consider the etiology of each arch. The literature indicates that in the majority of the cases in a healthy population, a cross-bite is usually adduced to a deficiency in jaw width [41], especially when unilateral [42]. Similarly, studies assessing craniofacial measurements of DS patients have suggested a maxillary hypoplasia, and a smaller maxilla characterized by a reduced anterior basal and apical dimension compared to age- and sex-matched healthy controls [43]. Other studies have also supported a small mandible compared to the general population [44]. As a result of small craniofacial bones, a prominent feature of DS patients is a relative macroglossia, rather than an absolute large tongue [45]. Other additional factors that may have contributed to the development of malocclusions and other orthodontic parameters (such as overjet and open bite) were not considered in this study, including obstructive sleep apnea, adenotonsillar hypertrophy, mouth breathing, breastfeeding and oral habits like thumb or finger sucking, among others [46,47,48]. The clinical outcomes investigated in the present study did not include the assessment of dental anomalies nor any cephalometric parameters as a radiographic examination (panoramic or lateral cephalogram) was not part of the study. This is attributed to the fact that the participants included in this study were seen in the dental clinic, but were not necessarily seeking or in need of treatment. As such, in order to prioritize achieving our expected sample size, the study was designed to spare the participants from any additional radiation that could potentially reduce the recruitment rate. Finally, as the study included only patients from the Italian population, the results of the present investigation cannot be generalizable to other ethnicities.

## 5. Conclusions

The present study showed that the prevalence of malocclusion is significantly and consistently higher in DS patients compared to non-syndromic controls, regardless of age. The most prevalent malocclusion in this population was a Class 3 molar relationship. Moreover, DS patients were more likely to exhibit a concave profile, anterior and posterior crossbite and maxillary transverse discrepancy. Some variability in the remaining orthodontic parameters could be observed according to the different age groups.

## Figures and Tables

**Table 1:** Demographics and comparison between DS and non-DS children in malocclusion and orthodontic parameters. Values are presented as percentage or as means ± standard deviation.

	DS Children (N = 15)	Non-DS Children (N = 15)	*p* Value
Age (years)	6.7 ± 2.0	7.0 ± 1.3	
Male/female, N	12/3	12/3	
Angle’s malocclusion			
Angle’s Class 1	42.9%	86.7%	0.021 *
Angle’s Class 2	7.1%	6.7%	0.742
Angle’s Class 3	50.0%	6.7%	0.014 *
Facial profile			
Straight	40.0%	93.3%	0.005 *
Convex	6.7%	0.0%	0.500
Concave	53.3%	6.7%	0.014 *
Tranverse discrepancy			
Maxillary transverse discrepancy	53.3%	26.7%	0.264
Posterior crossbite	21.4%	0.0%	0.100
Scissor bite	0.0%	0.0%	1.000
Sagittal discrepancy			
Anterior crossbite	35.7%	0.0%	0.017 *
Vertical discrepancy			
Open bite	21.4%	0.0%	0.100
Deep bite	13.3%	13.3%	1.000
Dental crowding	6.7%	6.7%	1.000
Probable sleep bruxism	60.0%	33.3%	0.272

DS = Down Syndrome; N = number. * identifies significant differences (*p* < 0.05).

**Table 2:** Demographics and comparison between DS and non-DS adolescents in malocclusion and orthodontic parameters. Values are presented as percentage or as means ± standard deviation.

	DS Adolescents (N = 23)	Non-DS Adolescents (N = 24)	*p* Value
Age (years)	13.1 ± 2.3	13.2 ± 1.9	
Male/female, N	11/12	13/11	
Angle’s malocclusion			
Angle’s Class 1	39.1%	58.3%	0.248
Angle’s Class 2	4.3%	26.0%	0.097
Angle’s Class 3	56.6%	16.7%	0.006 *
Facial profile			
Straight	47.8%	62.5%	0.385
Convex	4.3%	29.2%	0.048 *
Concave	47.8%	8.3%	0.003 *
Transverse discrepancy			
Maxillary transverse discrepancy	78.3%	16.7%	<0.001 *
Posterior crossbite	56.5%	4.2%	<0.001 *
Scissor bite	4.3%	4.2%	1.000
Sagittal discrepancy			
Anterior crossbite	43.5%	4.2%	0.002 *
Vertical discrepancy			
Open bite	26.1%	16.7%	0.494
Deep bite	13.0%	29.2%	0.286
Dental crowding	21.7%	16.7%	0.724
Probable sleep bruxism	43.5%	16.7%	0.060

DS = Down Syndrome; N = number. * identifies statistically significant differences (*p* < 0.05).

**Table 3:** Demographics and comparison between DS and non-DS adults in malocclusion and orthodontic parameters. Values are presented as percentage or as means ± standard deviation.

	DS Adults (N = 34)	Non-DS Adults (N = 36)	*p* Value
Age (years)	28.1 ± 9.0	28.3 ± 9.0	
Male/female, N	23/11	20/16	
Angle’s malocclusion			
Angle’s Class 1	18.8%	55.6%	0.003 *
Angle’s Class 2	21.9%	27.8%	0.780
Angle’s Class 3	59.4%	16.7%	<0.001 *
Facial profile			
Straight	29.4%	50.%	0.093
Convex	11.8%	36.1%	0.025 *
Concave	58.8%	13.9%	<0.001 *
Transverse discrepancy			
Maxillary transverse discrepancy	73.5%	33.3%	0.001 *
Posterior crossbite	22.2%	4.2%	0.001 *
Scissor bite	6.3%	2.8%	0.598
Sagittal discrepancy			
Anterior crossbite	31.3%	0.0%	<0.001 *
Vertical discrepancy			
Open bite	21.2%	5.6%	0.076
Deep bite	9.1%	27.8%	0.066
Dental crowding	23.5%	33.3%	0.433
Probable sleep bruxism	60.6%	22.2%	0.002 *

DS = Down Syndrome; N = number. * identifies statistically significant differences (*p* < 0.05).

## Data Availability

All of the data created and collected have been presented in the current study.
